# In vivo optical coherence tomography of experimental thrombosis in a rabbit carotid model

**DOI:** 10.1136/hrt.2007.117382

**Published:** 2007-10-18

**Authors:** L Meng, B Lv, S Zhang, B Yv

**Affiliations:** Department of Cardiology of the 2nd Affiliated Hospital of HarBin Medical University, HarBin, China

## Abstract

**Background:**

Plaque rupture with subsequent thrombosis is recognised as the underlying pathophysiology of most acute coronary syndromes. Thus, direct thrombus visualisation in vivo may be beneficial for both diagnosis and guidance of therapy. We sought to test the feasibility of imaging acute thrombosis in vivo using optical coherence tomography (OCT) in an experimental thrombosis animal model.

**Methods and results:**

Nine male New Zealand White rabbits (weight ≈3.0 kg) were made atherosclerotic with a high-cholesterol diet after injury of the right carotid artery endothelium. Thrombus was then induced with the use of Russell’s viper venom (RVV) and histamine. Subsequently, OCT imaging of the right carotid artery was performed. Histology was performed on arterial regions that were injured by balloon. Six rabbits (67%) developed thrombus. Histological correlation confirmed all thrombi (100%) detected with OCT, with no other thrombi seen in the other regions of the right carotid artery. In the remaining three rabbits, no thrombus was observed by OCT or histology.

**Conclusion:**

We demonstrate the feasibility of OCT for the detection of acute thrombosis in vivo using an animal model of atherosclerosis and acute thrombosis. Potential clinical applications include thrombus detection in acute coronary syndromes.

Acute cardiovascular events remain the leading cause of death worldwide.^1^ ^2^ The underlying mechanism of most acute coronary syndromes is the disruption of an atherosclerotic coronary plaque with an overlying thrombus.^3^^–^6 Although the accepted standard for the in vivo observation of this disease progression is currently coronary angiography, this gives little information regarding the composition of the atheromatous wall. Angiography also frequently fails to detect the presence of thrombus or differentiates thrombus from underlying atheroma.^7^ ^8^ Recent developments in other imaging methods offer alternatives for the in vivo detection and observation of this disease progression. Non-invasive imaging methods, such as magnetic resonance and computed tomography, are currently under intense investigation and development. Catheter-based imaging, however, can provide localised structural information with higher resolution than non-invasive imaging; therefore angioscopy and intravascular ultrasound (IVUS) are widely used in interventional cardiology. A recent publication suggests a correlation between the presence of lesion zones in coronary plaques appearing in angioscopy or IVUS images and acute coronary events.

Intravascular optical coherence tomography (OCT) has recently been proposed as a high-resolution imaging tool for plaque characterisation. Jang *et al*^9^ has proved that OCT images reveal additional morphological information beyond that in IVUS images, which can be used to improve plaque composition characterisation. OCT is a non-contact, light-based imaging method utilising newly developed fibreoptic technology. OCT gathers two-dimensional (2D) cross-sectional images from target tissues. In medical applications, it can be used to study tissues in vivo without having to excise the tissue from the patient. OCT employs near-infrared light, while conventional ultrasound imaging employs sound waves. The frequencies and bandwidths of infrared light are orders of magnitude higher than medical ultrasound signals, resulting in greater image resolution. The typical OCT image has a homoaxial resolution of 10 μm, which is 10 times higher than that of any clinically available diagnostic imaging method. Thereby, this technology provides in situ images of tissues at near histological resolution.

OCT has been shown to detect atheromatous plaques in the large arteries (experimental^10^^–^12 human^12^^–^17) and postmortem thrombosis,^18^ but has not yet been applied to detecting thrombosis in acute coronary syndromes in vivo. Using a modified Constantinides model of plaque disruption,^19^ ^20^ we sought to determine whether OCT could detect a thrombus overlying an atherosclerotic plaque, a frequent occurrence in plaque disruption.

## METHODS

### Rabbit housing and diet

**Figure 1:**
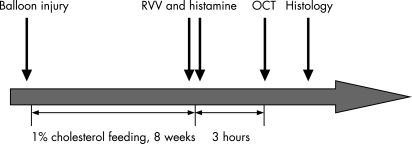
Schematic diagram of study time line. Balloon injury was followed by 8 weeks of 1% cholesterol feeding. OCT of the rabbit right common carotid artery was performed 3 hours after pharmacological triggering.

Nine adult male New Zealand White rabbits weighing 2.5–3 kg were continuously housed at the hospital’s (the 2nd Affiliated Hospital of HarBin Medical University) animal care facilities. All studies were performed under the approval of the hospital scientific affairs committee on animal research and ethics. The study protocol is summarised in figure 1. Animals were fed a 1% high-cholesterol diet incorporated into the rabbit chow (Purina modified 1% cholesterol diet 5736C-G) beginning immediately after balloon injury and continuing for 8 weeks.

### Balloon injury and endothelial denudation

Rabbits were anaesthetised with ketamine (5 mg/kg intramuscularly (IM)), xylazine (5 mg/kg IM) and acepromazine (0.75 mg/kg IM). Anaesthesia was maintained during the procedure with isoflurane inhalation via mask. Balloon-induced arterial wall injury of the right common carotid artery was performed with a 3F Fogarty catheter introduced through the arteria carotis externa of a right carotid artery cut-down. The catheter was first advanced 5 cm, to a level just above the right arteriae subclavia. The balloon was then inflated with 0.3 ml saline, and the catheter was gently retracted to the arteria carotis externa artery. This procedure was performed three times in succession in each rabbit. The catheter was then removed, and the incision was sutured closed.

### Pharmacological triggering and euthanasia

After endothelial denudation and 8 weeks of the high-cholesterol diet, plaque disruption was triggered by using Russell’s viper venom (RVV, Sigma Chemical Co) and histamine (Sigma Chemical Co) according to the method of Constantinides and Chakravarti.^21^ RVV (0.15 mg/kg) was given by intraperitoneal injection 3.5 hours before the rabbits were killed. Thirty minutes after each RVV injection, histamine (0.02 mg/kg) was administered intravenously through an ear vein. Then 3 hours after histamine injection OCT imaging of the right carotid artery was performed. Rabbits were killed after the OCT procedure using an overdose of intravenous sodium pentobarbital (100 mg/kg). The time course of the protocol is shown in figure 1.

### OCT imaging

Rabbits were sedated by using ketamine (35 mg/kg IM), xylazine (5 mg/kg IM), and acepromazine (0.75 mg/kg IM) for 3 hours after pharmacological triggering. An intravascular OCT imaging catheter (ImageWire, LightLab Imaging, Westford, MA, USA) was inserted sequentially through the cut-down of the right carotid artery. A scout image of the right carotid artery was used to position the OCT ImageWire within the right carotid artery at the distal carotid artery bifurcation. Serial images of OCT were obtained in an automated pull-back format at a rate of 1 mm/s and 15 frames/s during intermittent saline flush a through the guiding catheter to transiently displace blood.

The appearance of thrombus in OCT images was interpreted to be a signal-reflecting region detached from the vessel wall and protruding into the signal-poor vessel lumen. OCT images were processed and analysed with NIH Image (public domain software by Dr Wayne Rasband, National Institutes of Health, Bethesda, MD, USA). The ability of OCT to visualise thrombi was compared with histological diagnosis, which served as the “gold standard”.

### Tissue preparation

After euthanasia of the animals, the heart was removed and perfusion-fixed by using 10% formalin acetate for a minimum of 2 hours. The right carotid artery, from the cutdown to the bifurcation, was removed, cut into 3.5-mm serial sections and catalogued. These samples then underwent additional fixation overnight in formalin, followed by overnight tissue processing and dehydration. The samples were embedded in paraffin the following day. The serial cross-sections were processed for general histological staining with haematoxylin and eosin (HE) and assessed visually for the presence or absence of thrombus. When detected, thrombus type was classified into either red or white by histological examination. Histological cross-sections were then compared with the corresponding OCT cross-sectional images using the right carotid artery bifurcation as the common landmark for localisation.

### Statistical analysis

All data are reported as mean (SD). Comparisons and correlations were made by linear regression and Fisher’s exact test, where appropriate. A value of p⩽0.05 was considered significant.

## RESULTS

All animals underwent initial balloon injury without complications and recovered uneventfully. The weight of the rabbits at baseline was 2.7 (0.2) kg and increased to 2.9 (0.3) kg (p = 0.13) after the 8-week 1% cholesterol diet period.

### Rate of thrombus formation

**Figure 2:**
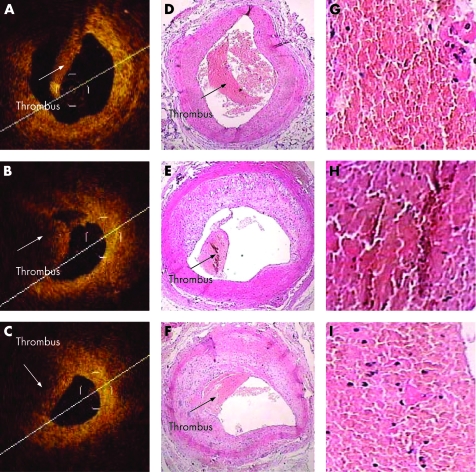
Comparative analysis of the histomorphometric and OCT features of carotid artery thrombus presence or absence of thrombus. Correlation between OCT images obtained in vivo and later histological examination of animal model red thrombi. A, B and C show OCT images of the right common carotid artery cross-sections, with each showing a signal-rich mass protruding into the lumen. The corresponding histological sections, D, E and F, confirm the thrombus within the lumen of the common carotid artery at these levels. OCT images of red thrombi are characterised as high-backscattering protrusions with signal-free shadowing (arrows). Red thrombus, which is a cell-rich structure and consists mainly of red blood cells, causes scatter and attenuation of OCT signal intensity from the inner surface of the thrombus to the vessel wall. OCT was able to determine the presence or absence of a thrombus in all arterial segments.

Just before euthanasia of the animals, six (67%) had visible evidence of a mass protruding into the vessel lumen in the OCT images. All nine rabbits had evidence of atherosclerosis on histology, as indicated by intimal thickening and foam cells. Red thrombi were found by histological examination in 12 (13%) of the 90 carotid artery samples. Representative OCT images of red thrombi and their corresponding histological images are shown in figure 2.

### Location and length of thrombus

**Figure 3:**
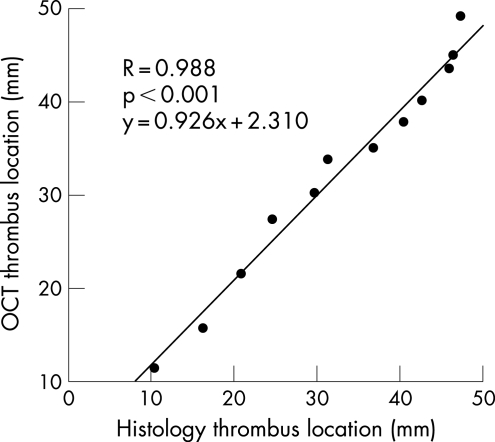
Linear regression analysis comparing the location of the most distal point of each thrombus as measured from the distal carotid artery bifurcation, as localised by OCT and histopathology.

**Figure 4:**
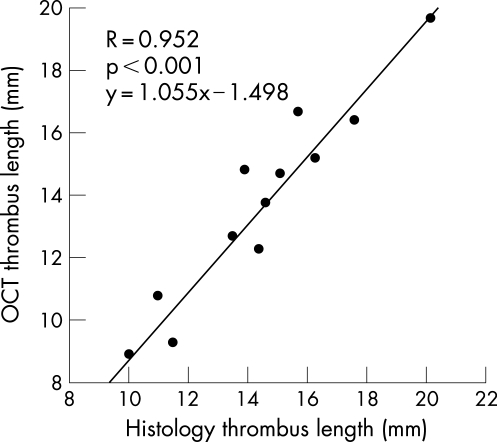
Linear regression analysis comparing thrombus length, as measured by OCT and histopathology.

The thrombus location, defined as the most distal point of the thrombus from the distal carotid artery bifurcation, showed excellent correlation (R = 0.988, p<0.001) between histology and OCT (fig 3). Similarly, there was very good correlation (R = 0.952, p<0.001) between thrombus length by OCT and histology (fig 4).

## DISCUSSION

This is the first in vivo study of detailed carotid artery thrombus morphology in an atherosclerotic rabbit model of thrombosis. In the present study, intravascular OCT was shown to accurately identify new thrombi in vivo in rabbits that had undergone pharmacological triggering. There was also excellent correlation between OCT images and histology regarding thrombus length and location.

Compared with previous postmortem studies by Kume *et al*,^18^ we imaged acute thrombi by means of OCT in an in vivo animal atherosclerosis model of thrombosis after pharmacological triggering. The combination of balloon injury and cholesterol feeding, as was used in our modified Constantinides model, resulted in lesions that are more uniform in size and distribution and produced plaques resembling those found in human coronary arteries.^22^ This animal model reproducibly produces in vivo thrombi at a rate of more than 60%.

Recently, there has been considerable interest in the use of OCT for the assessment of atherosclerosis because it can evaluate the plaque directly in vivo and can potentially determine whether a plaque is vulnerable to disruption.^10^ Zimarino *et al*^11^ ^12^ and others^13^^–^16 have performed OCT studies to characterise atherosclerosis ex vivo in human plaques and in vivo in animal models. Kume *et al*^18^ detected red and white coronary arterial thrombi by using OCT ex vivo in postmortem patients, but did not show thrombus images in vivo. This study sought to further investigate the ability of OCT to detect thrombus in vivo, and to render the distinction between thrombi and plaques more easily and more reliably. Our results confirm the findings of Kume *et al*^18^ as to the detection and evaluation of red thrombus. The appearance of red thrombus in this study agreed with that in the previous postmortem study. Although we did not observe any instance of white thrombus in this study, we predict that a significant difference in attenuation of OCT light intensity between red and white thrombi would have been observed in images acquired in vivo. Altogether the differentiation of different histological types of thrombi by OCT is with high sensitivity and specificity, as shown by Kume *et al*.^18^ (This hypothesis may be our next research project.)

### Potential clinical applications

Patients experiencing acute coronary syndromes may present with chest pain/symptoms but lack the diagnostic ECG changes when in emergency triage. Early detection of white or red thrombus via OCT imaging may be beneficial for both diagnosis and early treatment of acute coronary syndromes.

### Limitations of the study

There are limitations to the present study. All the thrombi found in our study were red thrombi consisting mainly of red blood cells, which are similar to acute artery thrombi in clinical scenarios. Therefore further studies should evaluate more time points in order to image white thrombi so as to distinguish the differences in appearance between red and white thrombus in vivo in OCT images.

An inherent limitation of most light-based imaging methods is the need to achieve a blood-free imaging zone, which in this study was achieved through intermittent saline flushes through the coronary guide catheter. By using such a flushing technique, neither evaluation of different types of thrombi nor estimation of plaque morphology with OCT imaging is possible in a human clinical scenario.^17^ Even utilising this flushing technique in this animal model, the image acquisition time was limited to only a few seconds, preventing pull-back imaging of long arterial segments. A further limitation of OCT technology is the relatively shallow axial penetration (2 mm in a blood-free environment) of the signal into thrombi or tissue. As a result, it is possible that this study failed to image through the entire dimension of any thrombi larger than 2 mm in thickness.

## CONCLUSIONS

In this rabbit carotid artery model of acute thrombosis after pharmacological triggering, we demonstrate the ability of in vivo OCT imaging to determine the presence, location and size of a thrombus. The combination of in vivo OCT and the modified Constantinides animal model may be an important research tool in furthering our understanding and treatment of acute coronary syndromes accompanied by thrombus.
